# Supplementary material for: Orphan quality control by an SCF ubiquitin ligase directed to pervasive C-degrons
Source: Nat Commun. 2023 Dec 15;14:8363. doi: 10.1038/s41467-023-44096-z (PMC10724198; doi:10.1038/s41467-023-44096-z)
Supplement: Supplementary file 1 — Supplementary Information [file 41467_2023_44096_MOESM1_ESM.pdf]

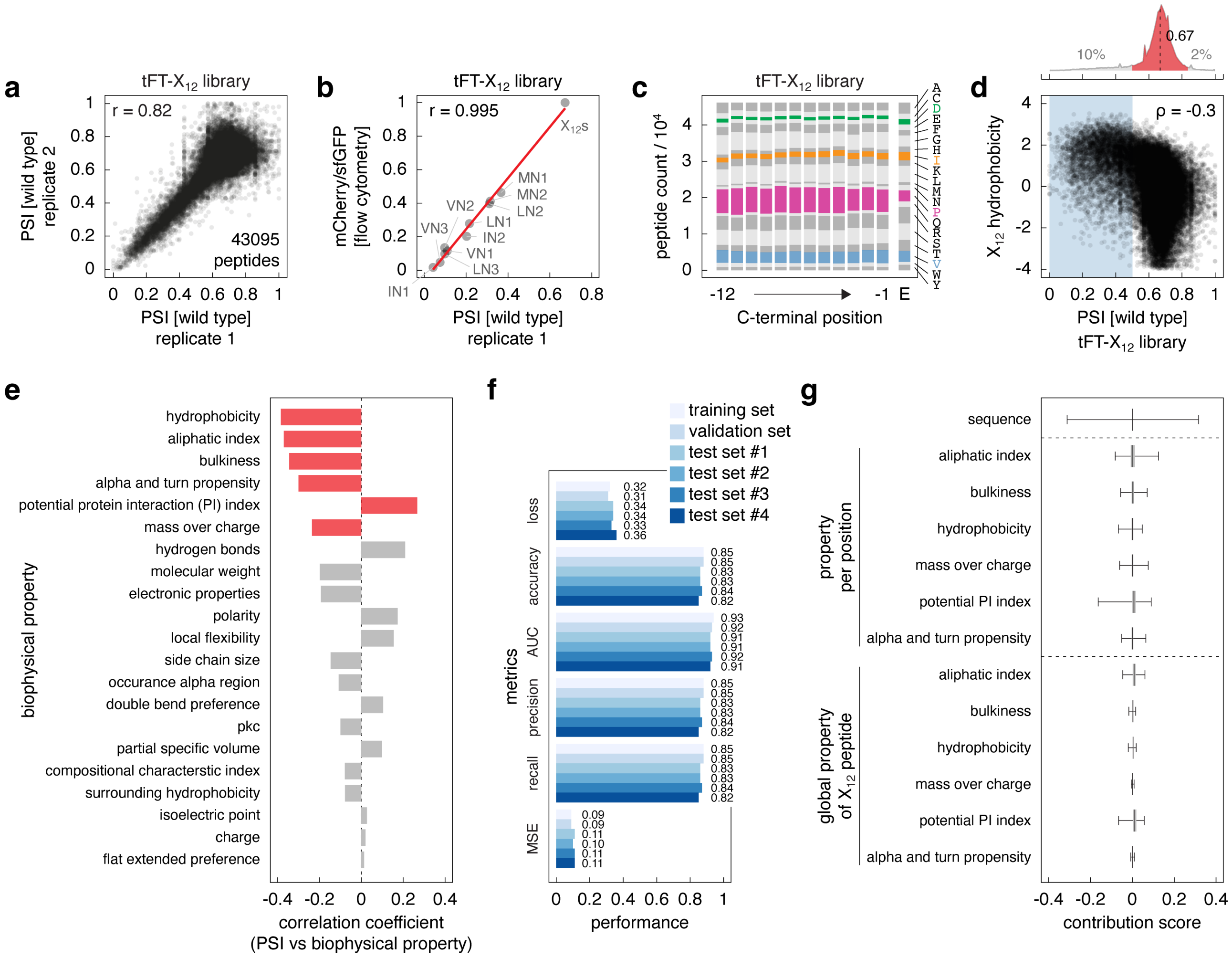

Supplementary Figure 1. Search for C-degrons using random peptide libraries.

a – Reproducibility of MPS profiling. PSIs in the tFT-X_{12} library from two independent MPS profiling experiments. Only peptides with at least 10 sequencing reads in both replicates were included in the comparison. r, pearson correlation coefficient.

b – Correlation between PSI in the tFT-X_{12} library and mCherry/sfGFP ratios. Strains expressing the indicated constructs were analyzed with flow cytometry (Supplementary Fig. 3d).

c – Amino acid frequency in the tFT-X_{12} library. Number of X_{12} peptides with each amino acid per C-terminal position. The expected distribution (E) based on equal frequency of all codons is shown for comparison.

d – Definition of the PSI threshold separating unstable from stable constructs. Top, distribution of PSIs in replicate 1 of the tFT-X_{12} library. The median ± 3.7 x median absolute deviation range (Methods) is highlighted in red. The percentage of PSIs outside of this range is indicated. Bottom, correlation between peptide hydrophobicity and PSI in the tFT-X_{12} library from Fig. 1e. ρ, spearman correlation coefficient.

e – Pearson correlation coefficients between PSI and global biophysical properties for the 46152 peptides in the tFT-X_{12} library, replicate 1. Red, biophysical properties with highest absolute correlations included in the deep neural network model from Fig. 2a.

f – Performance metrics for the deep neural network model from Fig. 2a, trained on the tFT-X_{12} library to classify peptides into stable or unstable groups. AUC, area under the receiver operating characteristic curve; MSE, mean squared error (Methods).

g – Distributions of SHAP contribution scores for 4110 peptides classified as unstable by the deep neural network model from Fig. 2a. Centerlines mark the medians, box limits indicate the 25th and 75th percentiles, and whiskers extend to the minimum and maximum value in each group.

Source data are provided as a Source Data file.

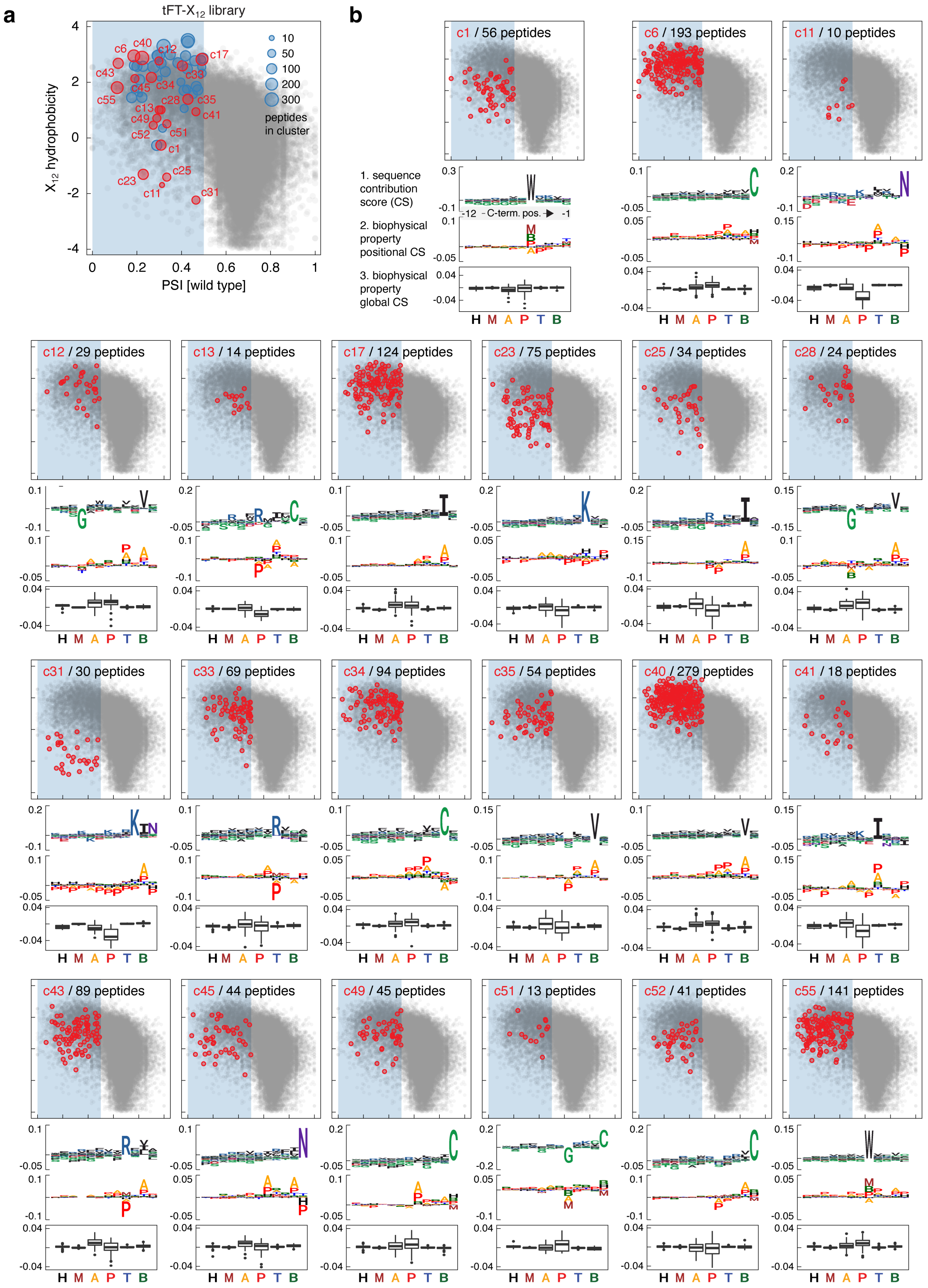

Supplementary Figure 2. C-terminal motifs identified in the tFT-X_{12} degrons.

a – Distribution of putative degrons in the tFT-X_{12} library into 56 groups by k-means clustering of vectors of SHAP contribution scores, as in Fig. 2b. Clusters with at least one mean sequence contribution score > 0.05 at positions -5 to -1 are highlighted (Methods).

b – Description of clusters highlighted in **a**. Top, hydrophobicity (y-axis) and PSI (x-axis) for all peptides in a cluster (axes as in **a**), compared to all peptides in the tFT-X_{12} library (grey). Bottom, mean SHAP contribution scores of the peptides in the cluster, as exemplified in Fig. 2a. In the box plots, centerlines mark the medians, box limits indicate the 25th and 75th percentiles, and whiskers extend to 1.5xIQR (interquartile range) in each group. Axis units and labels are omitted for simplicity.

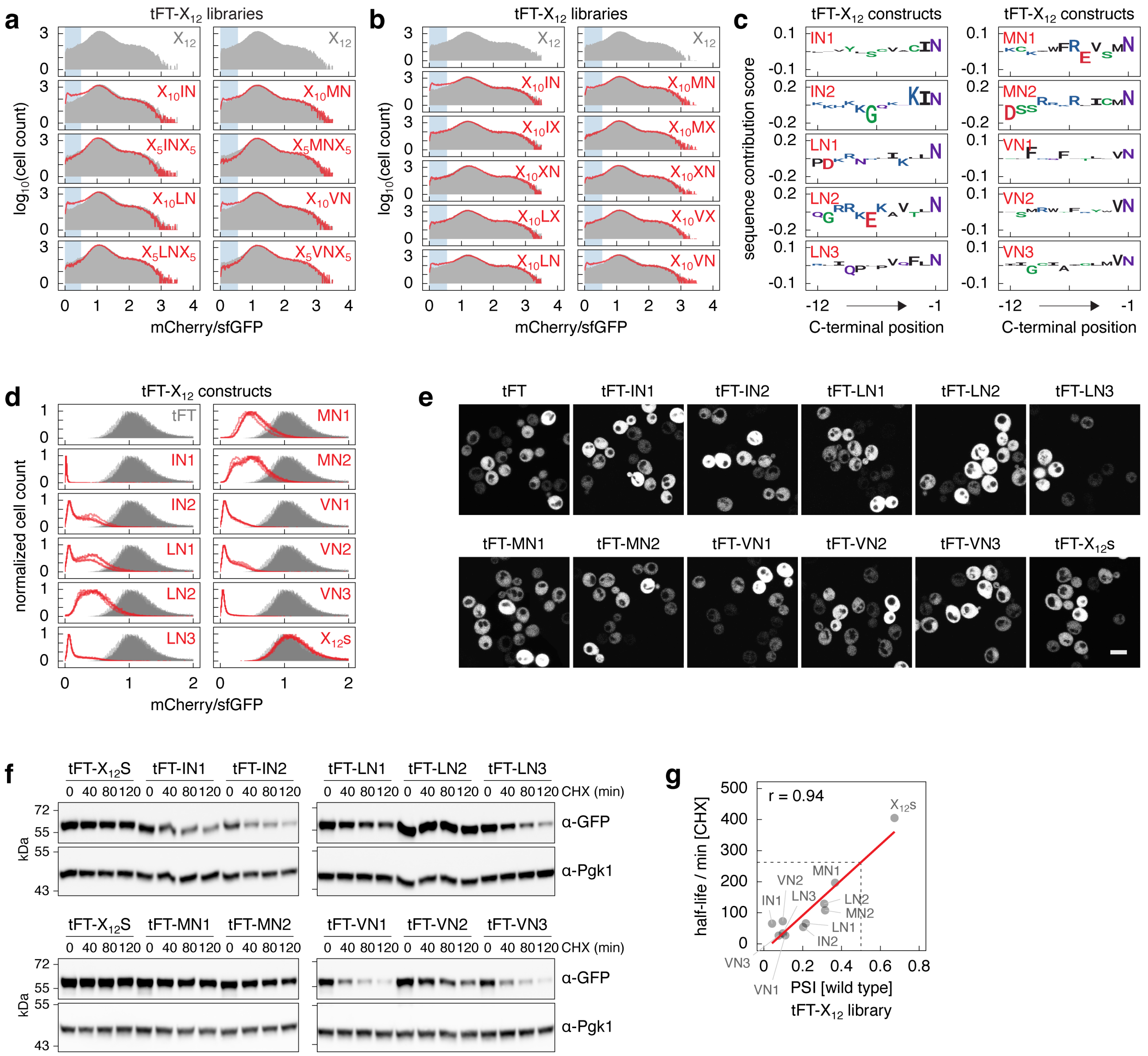

Supplementary Figure 3. [ILMV]N C-termini promote protein degradation.

a, b – Distributions of mCherry/sfGFP ratios, determined by flow cytometry, in pooled yeast libraries expressing tFT-tagged random peptides 12 amino acids in length and with the indicated fixed positions. The distribution in the tFT-X_{12} library is shown for comparison (grey). N = 10^5 cells per sample. Blue regions mark cells expressing unstable variants (mCherry/sfGFP < 0.5). This threshold is more stringent than the PSI threshold used to define degrons in MPS profiling (PSI < 0.5) (Fig. 1c, e). For comparison, the stable reference construct tFT-X_{12}s has a PSI of 0.67 (Supplementary Fig. 1b) and a median mCherry/sfGFP ratio of 1.3 in this flow cytometry analysis.

c – SHAP sequence contributions scores for the putative ΦN C-degrons from Fig. 3a, generated using the workflow outlined in Fig. 2a.

d – Distributions of mCherry/sfGFP ratios, determined by flow cytometry, of strains expressing tFT-X_{12} constructs. A strain expressing only the tFT is shown for comparison (grey). n = 4, > 20000 cells per replicate.

e – Fluorescence microscopy of strains expressing the indicated tFT-X_{12} constructs. Representative examples of sfGFP fluorescence. Scale bar, 5 µm.

f – Degradation of tFT-X_{12} constructs after blocking translation with CHX. Whole-cell extracts were separated by SDS-PAGE, followed by immunoblotting with antibodies against GFP and Pgk1 as the loading control.

g – Correlation between PSI (profiling of the tFT-X_{12} library, Fig. 1e) and half-life (determined with CHX chases, Fig. 3c) for the tFT-X_{12} constructs in Fig. 3a. A PSI of 0.5 corresponds to a half-life of 262 min (dashed lines) based on the linear fit in red.

Source data are provided as a Source Data file.

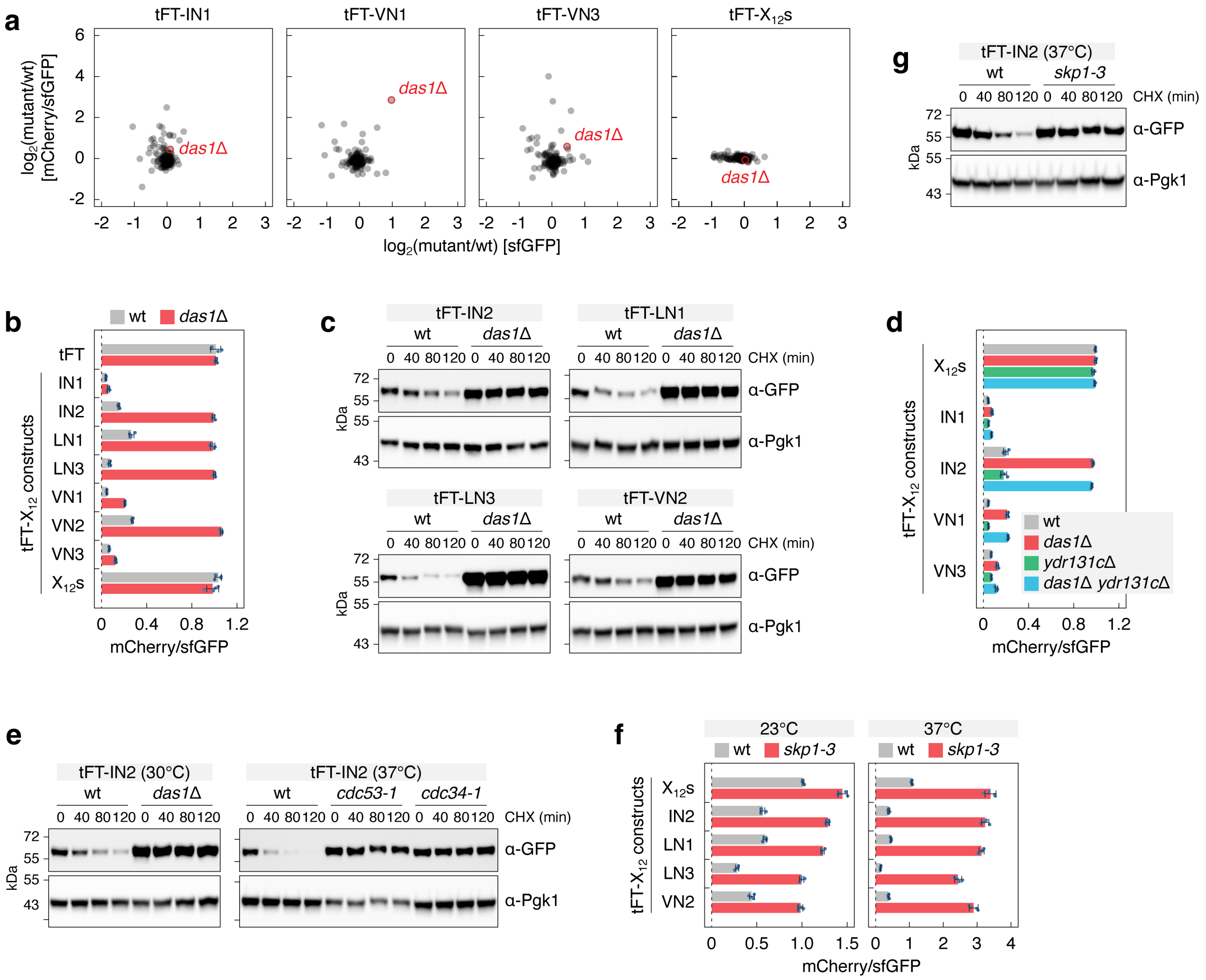

Supplementary Figure 4. Degradation of proteins with [ILMV]N C-degrons depends on SCF^{Das1}.

a – Targeted screens to identify UPS factors involved in turnover of the indicated tFT-X_{12} constructs, performed according to Fig. 3d. Mean log_2 fold changes (n = 4) in sfGFP intensity and mCherry/sfGFP ratio between each mutant and a wild type control (wt, *his3*Δ::kanMX).

b, d, f – mCherry/sfGFP ratios of colonies expressing tFT-X_{12} constructs or the tFT alone for comparison (mean ± s.d., n = 4). Measurements at permissive (23°C) or restrictive (37°C) temperatures for the *skp1-3* allele and the corresponding wild type (**f**).

c, e, g – Degradation of tFT-X_{12} constructs after blocking translation with CHX. Whole-cell extracts were separated by SDS-PAGE, followed by immunoblotting with antibodies against GFP and Pgk1 as the loading control. Strains were shifted to 37°C for 3 h before adding CHX.

Source data are provided as a Source Data file.

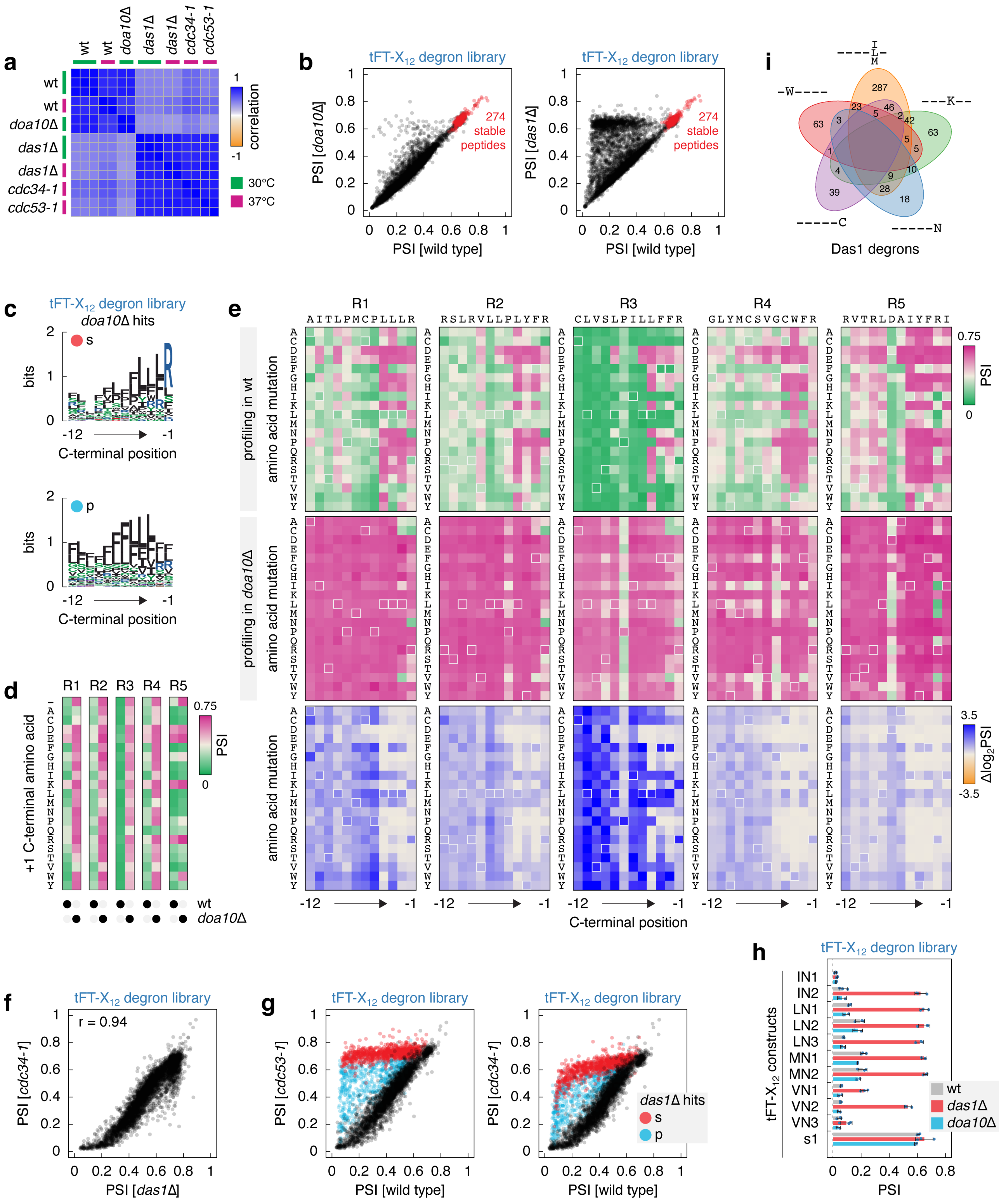

Supplementary Figure 5. Survey of C-degrons targeted by the SCF^{Das1} ubiquitin ligase.

a – Heatmap of pearson correlation coefficients between profiles of PSIs determined by MPS profiling of the tFT-X_{12} degron library (Fig. 5a) in the indicated genetic backgrounds.
b – PSIs in the tFT-X_{12} degron library determined by MPS profiling in the indicated genetic backgrounds. Stable control peptides are indicated.
c – Sequence logos for the tFT-X_{12} constructs fully stabilized (s, top) or partially stabilized (p, bottom) in the *doa10Δ* mutant (Fig. 5b).
d, e – Heatmaps of PSIs determined by MPS profiling. Impact of extending the C-terminus by a single amino acid (+1 position) (**d**) and saturation mutagenesis (**e**) of tFT-R1, tFT-R2, tFT-R3, tFT-R4 and tFT-R5 constructs

from Fig. 5b. Heatmaps of differences in PSI between the saturation mutagenesis libraries in the two genetic backgrounds (log_2PSI[*das1*Δ]-log_2PSI[wt]) (**e**, bottom).
f, g – PSIs in the tFT-X_{12} degron library, excluding the stable control peptides, determined by MPS profiling in the indicated genetic backgrounds. Constructs fully stabilized (s) or partially stabilized (p) in the *das1*Δ mutant are indicated (**g**).
h – PSIs of the indicated constructs determined by MPS profiling of the tFT-X_{12} degron library in the wild type, *das1*Δ and *doa10*Δ backgrounds at 30°C. A stable control peptide, s1 (ESCWVSRVGVCR), is shown for comparison (mean ± s.d., n = 2 (*doa10*Δ) or 3).
i – Venn diagram of the 653 Das1 degrons in Fig. 5k matching the five potential degron motifs.
Source data are provided as a Source Data file.

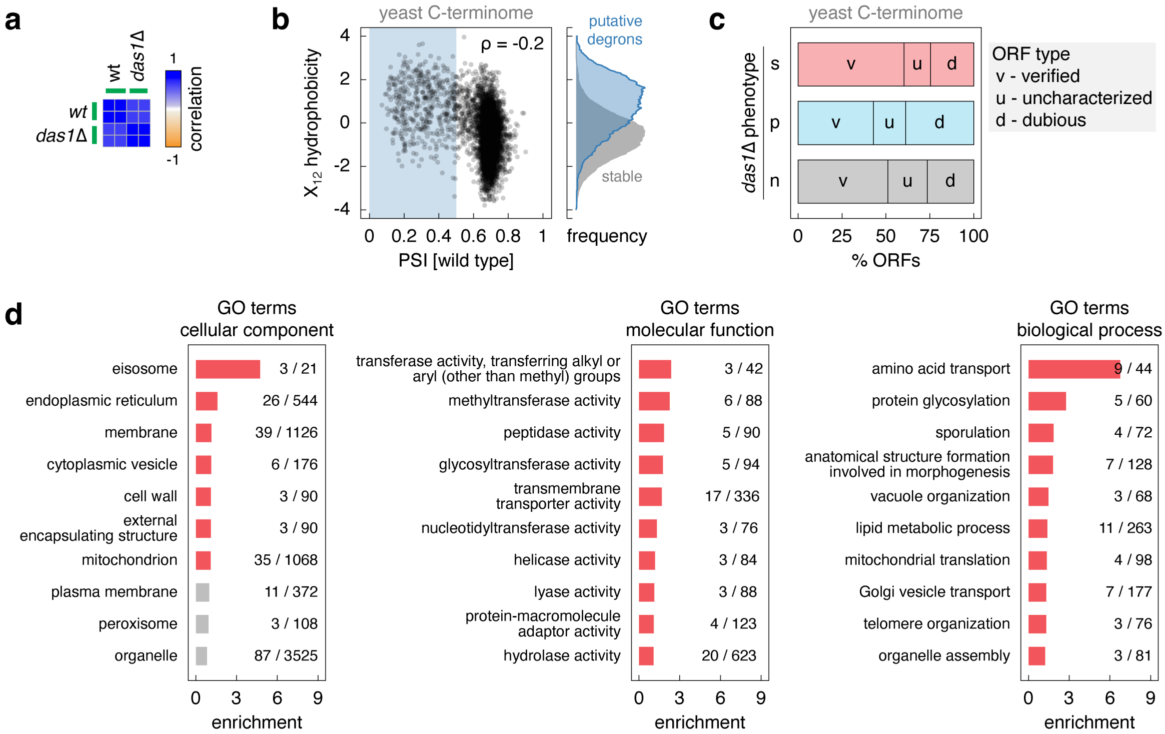

e

protein	C-terminus	complex	PMID
Atg1	LKILRQKMNHQN	Atg1-Atg13-Atg17	15743910
Dug3	RTRPPTAVAHIA	Dug2-Dug3	20620961
HO	KSCGNNAGARIC		
Irc4	VPEEQRIKRRKI		
Pex12	WEVVTGIRKLLI	Pex2-Pex10-Pex12	12667447
Rer2	GDPIDLKEKKLN	Nus1-Rer2	25066056
Rfc2	QLLNLLVKISQL	Elg1 RFC-like	20620961
Rfs1	QGKAFYDRIKDL	putative Ycp4-Rfs1-Pst2	20620961
Rlp7	EVDIDSLLAKLN	preribosome, large subunit precursor	20620961
Rpa12	CTSCGYKFRTNN	RNA polymerase I	20620961
Rpo31	ESLSNEAALKAN	RNA polymerase III	20620961
Rtt107	SKKNVLYQKKNN	CUL8-RING ubiquitin ligase	20139071
Shc1	TSHEAVAAKKLI		
Swd1	TDYTRIIKMQSS	COMPASS	20620961
Ubp8	AYLLFYTIRQVN	SAGA	20620961
Ybl044w	RGSCDNWKKRQN		
Ycl049c	TLKKIFSKRSIS		
Yfl012w	RDLNKLRSFQNF		

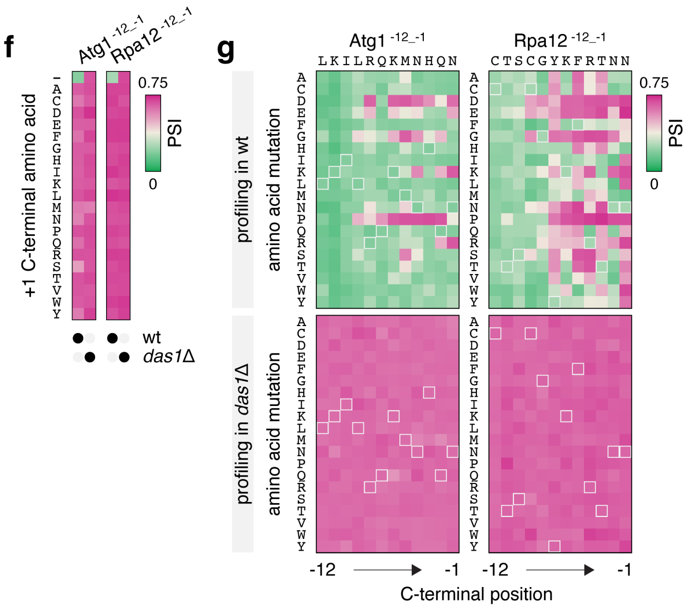

Supplementary Figure 6. Substrates of the SCF^{Das1} ubiquitin ligase.

a – Heatmap of pearson correlation coefficients between profiles of PSIs determined by MPS profiling of the yeast C-terminome library (Fig. 6a) in wild type and *das1*Δ backgrounds.
b – Correlation between peptide hydrophobicity and PSI in the yeast C-terminome library. Blue region marks putative degrons. Hydrophobicity distributions for the putative degrons and stable peptides (right).
c – Stratification of unstable constructs in the yeast C-terminome library (Fig. 6b) by type of ORF from which the C-terminal peptides originated. Constructs fully stabilized (s), partially stabilized (p) or not affected (n) in the *das1*Δ mutant are indicated.
d – Mapping of ORFs corresponding to the 198 Das1 C-degrons in Fig. 6b to gene ontology (GO) slim terms. For each GO term, the number of ORFs in the set of Das1 C-degrons and the number of ORFs in the yeast genome are indicated. Only the top 10 GO terms by enrichment are shown: red – enrichment, grey – no enrichment in the set of Das1 C-degrons.
e – Proteins with Das1-dependent turnover from Fig. 6f. PMID, PubMed unique identifiers of publications with annotations of the indicated complexes.
f, g – Heatmaps of PSIs determined by MPS profiling. Impact of extending the C-terminus by a single amino acid (+1 position) (**f**) and saturation mutagenesis (**g**) of tFT-Atg1$^{-12_-1}$ and tFT-Rpa12$^{-12_-1}$ constructs.

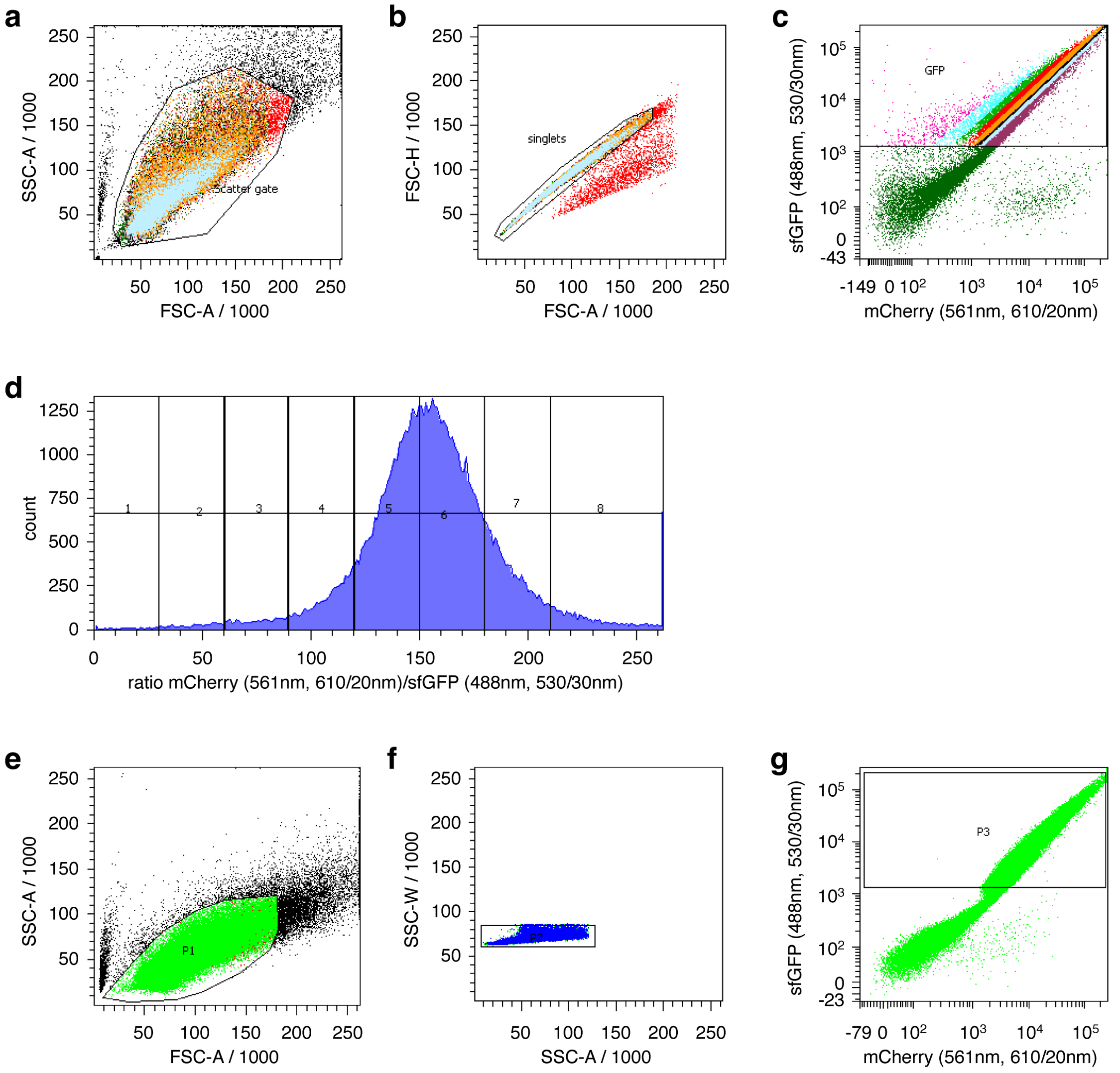

Supplementary Figure 7. Representative gating strategies used in FACS and flow cytometry experiments.

a-d – Representative gating strategy used for fluorescence-activated cell sorting in MPS profiling experiments, exemplified with the tFT-X_{12} library. Gated cells (**a**) were subsequently gated for single cells (**b**), followed by gating for GFP-fluorescent cells (**c**). The resulting population was sorted into 8 bins according to the mCherry/sfGFP ratio (**d**). The 8 bins are also indicated with different colors in **c**.

e-g – Representative gating strategy used in flow cytometry experiments, exemplified with a strain expressing the tFT-X_{12}s construct. Gated cells (**e**) were subsequently gated for single cells (**f**), followed by gating for GFP-fluorescent cells (**g**).

Supplementary Table 1. Yeast strains.

Strain	Genotype	Source
ESM356-1	MATa ura3-52 leu2Δ1 his3Δ200 trp1Δ63	Elmar Schiebel
BY4741	MATa his3Δ1 leu2Δ0 met15Δ0 ura3Δ0	Ref 1
Y8205	MATalpha can1Δ::STE2pr-SpHIS5 lyp1Δ::STE3pr-LEU2 his3Δ1 leu2Δ0 ura3Δ0	Ref 2
Y7092	MATalpha lyp1Δ his3Δ1 leu2Δ0 ura3Δ0 met15Δ0 can1Δ::STE2pr-SpHIS5	Ref 2
yMaM1205	Y7092 can1Δ::STE3pr-LEU2-GAL1pr-NLS-I-SCEI	Ref 3
PJ69-4A	MATa trp1-901 leu2-3,112 ura3-52 his3-200 gal4Δ gal80Δ LYS2::GAL1-HIS3 GAL2-ADE2 met2::GAL7-lacZ	Ref 4
yKEK430	Y8205 ura3Δ0::GPDpr-sfGFP-mCherry-IN1-URA3	this study
yKEK431	Y8205 ura3Δ0::GPDpr-sfGFP-mCherry-IN2-URA3	this study
yKEK432	Y8205 ura3Δ0::GPDpr-sfGFP-mCherry-LN1-URA3	this study
yKEK433	Y8205 ura3Δ0::GPDpr-sfGFP-mCherry-LN3-URA3	this study
yKEK436	Y8205 ura3Δ0::GPDpr-sfGFP-mCherry-VN1-URA3	this study
yKEK437	Y8205 ura3Δ0::GPDpr-sfGFP-mCherry-VN2-URA3	this study
yKEK438	Y8205 ura3Δ0::GPDpr-sfGFP-mCherry-VN3-URA3	this study
yKEK429	Y8205 ura3Δ0::GPDpr-sfGFP-mCherry-X_{12}s-URA3	this study
UPS array	BY4741 orfΔ::natNT2	this study (Supplementary Data 2)
yKEK451	BY4741 das1Δ::kanMX6	this study
cdc53-1	BY4741 cdc53-1::kanMX6 (from ts_v6 array)	Ref 5
cdc34-1	BY4741 cdc34-1::kanMX6 (from ts_v6 array)	Ref 5
skp1-3	BY4741 skp1-3::kanMX6 (from ts_v6 array)	Ref 5
yKEK476	PJ69-4A das1Δ::hphNT1	this study
yKEK452	ESM356-1 das1Δ::kanMX6	this study
YBB1	ESM356-1 doa10Δ::hphNT1	Ref 6
yKEK454	BY4741 ydr131cΔ::hphNT1	this study
yKEK456	yKEK451 ydr131cΔ::hphNT1	this study
yKEK457	yMaM1205 his3Δ::hphNT1	this study
yKEK458	yMaM1205 das1Δ::hphNT1	this study
N-SWAT library	BY4741 URA3-N_SWAT-sfGFP-ORF	Ref 7
yKEK491	BY4741 natNT2-GPDpr-ATG13	this study
yKEK492	BY4741 natNT2-GPDpr-ATG17	this study
yKEK494	BY4741 natNT2-GPDpr-RPA135	this study
yKEK495	BY4741 natNT2-GPDpr-RPA190	this study
yKEK513	yKEK491 das1Δ::hphNT1	this study
yKEK515	yKEK492 das1Δ::hphNT1	this study
yKEK517	yKEK494 das1Δ::hphNT1	this study
yKEK519	yKEK495 das1Δ::hphNT1	this study

Supplementary Table 2. Plasmids.

Plasmid	Description	Source
pKEK077	pRS413-GPDpr	Ref 8
pNS002	pRS413-GPDpr-sfGFP-mCherry-EcoRV	Ref 9
pKEK401	pRS413-GPDpr-sfGFP-mCherry-IN1	this study
pKEK402	pRS413-GPDpr-sfGFP-mCherry-IN2	this study
pKEK403	pRS413-GPDpr-sfGFP-mCherry-LN1	this study
pKEK404	pRS413-GPDpr-sfGFP-mCherry-LN2	this study
pKEK405	pRS413-GPDpr-sfGFP-mCherry-LN3	this study
pKEK406	pRS413-GPDpr-sfGFP-mCherry-MN1	this study
pKEK407	pRS413-GPDpr-sfGFP-mCherry-MN2	this study
pKEK408	pRS413-GPDpr-sfGFP-mCherry-VN1	this study
pKEK409	pRS413-GPDpr-sfGFP-mCherry-VN2	this study
pKEK410	pRS413-GPDpr-sfGFP-mCherry-VN3	this study
pKEK411	pRS413-GPDpr-sfGFP-mCherry-X12s	this study
pNS001	pRS413-GPDpr-sfGFP-mCherry	this study
pGADCg	Vector for C-terminal tagging of AD	Addgene #20161
pKEK412	pGADCg-Das1-AD	this study
pGBDC1	Vector for N-terminal tagging of DBD	Helle Ulrich
pKEK413	pGBDC1-DBD-DHFR-IN1	this study
pKEK414	pGBDC1-DBD-DHFR-IN2	this study
pKEK415	pGBDC1-DBD-DHFR-LN1	this study
pKEK416	pGBDC1-DBD-DHFR-LN3	this study
pKEK417	pGBDC1-DBD-DHFR-VN1	this study
pKEK418	pGBDC1-DBD-DHFR-VN2	this study
pKEK419	pGBDC1-DBD-DHFR-VN3	this study
pKEK420	pGBDC1-DBD-DHFR-X12s	this study
pKEK421	pRS413-GPDpr-sfGFP-mCherry-IN2(+1L)	this study
pKEK422	pRS413-GPDpr-sfGFP-mCherry-IN2(+1W)	this study
pKEK423	pRS413-GPDpr-sfGFP-mCherry-IN2(-1K)	this study
pKEK424	pRS413-GPDpr-sfGFP-mCherry-IN2(-1W)	this study
pKEK425	pRS413-GPDpr-sfGFP-mCherry-IN2(-1C)	this study
pKEK426	pGBDC1-DBD-DHFR-IN2(+1L)	this study
pKEK427	pGBDC1-DBD-DHFR-IN2(+1W)	this study
pKEK428	pGBDC1-DBD-DHFR-IN2(-1K)	this study
pKEK429	pGBDC1-DBD-DHFR-IN2(-1W)	this study
pKEK430	pGBDC1-DBD-DHFR-IN2(-1C)	this study
pSD-N1	N-SWAT donor plasmid for insertion of the desired tag	Ref 10
pKEK431	pSD-N1-HA	this study
pKEK432	pSD-N1-GPDpr-HA	this study
pKEK433	pSD-N1-mNG-mCherry	this study
pKEK434	pSD-N1-GPDpr-mNG-mCherry	this study
pKEK435	pRS413-GPDpr-sfGFP-mCherry-Atg1-12_-1	this study
pKEK436	pRS413-GPDpr-sfGFP-mCherry-Atg1-12_-1(+1L)	this study
pKEK437	pRS413-GPDpr-sfGFP-mCherry-Atg1-12_-1(-1K)	this study
pKEK438	pRS413-GPDpr-sfGFP-mCherry-Rpa12-12_-1	this study
pKEK439	pRS413-GPDpr-sfGFP-mCherry-Rpa12-12_-1(+1L)	this study
pKEK440	pRS413-GPDpr-sfGFP-mCherry-Rpa12-12_-1(-1K)	this study
pKEK441	pRS413-GPDpr-sfGFP-mCherry-Atg1	this study
pKEK442	pRS413-GPDpr-sfGFP-mCherry-Atg1(+1L)	this study
pKEK443	pRS413-GPDpr-sfGFP-mCherry-Atg1(-1K)	this study
pKEK444	pRS413-GPDpr-sfGFP-mCherry-Rpa12	this study
pKEK445	pRS413-GPDpr-sfGFP-mCherry-Rpa12(+1L)	this study
pKEK446	pRS413-GPDpr-sfGFP-mCherry-Rpa12(-1K)	this study

Supplementary References

1. Brachmann, C.B., Davies, A., Cost, G.J., Caputo, E., Li, J., Hieter, P., and Boeke, J.D. (1998). Designer deletion strains derived from Saccharomyces cerevisiae S288C: a useful set of strains and plasmids for PCR-mediated gene disruption and other applications. Yeast *14*, 115–132. 10.1002/(SICI)1097-0061(19980130)14:2<115::AID-YEA204>3.0.CO;2-2.

2. Tong, A.H.Y., and Boone, C. (2007). High-throughput strain construction and systematic synthetic lethal screening in Saccharomyces cerevisiae. Methods in Microbiology *36*. 10.1016/S0580-9517(06)36016-3.

3. Meurer, M., Duan, Y., Sass, E., Kats, I., Herbst, K., Buchmuller, B.C., Dederer, V., Huber, F., Kirrmaier, D., Štefl, M., et al. (2018). Genome-wide C-SWAT library for high-throughput yeast genome tagging. Nat Methods *15*, 598–600. 10.1038/s41592-018-0045-8.

4. James, P., Halladay, J., and Craig, E.A. (1996). Genomic libraries and a host strain designed for highly efficient two-hybrid selection in yeast. Genetics *144*, 1425–1436. 10.1093/GENETICS/144.4.1425.

5. Li, Z., Vizeacoumar, F.J., Bahr, S., Li, J., Warringer, J., Vizeacoumar, F.S., Min, R., VanderSluis, B., Bellay, J., Devit, M., et al. (2011). Systematic exploration of essential yeast gene function with temperature-sensitive mutants. Nat Biotechnol *29*, 361–367. 10.1038/nbt.1832.

6. Kats, I., Khmelinskii, A., Kschonsak, M., Huber, F., Knieß, R.A., Bartosik, A., and Knop, M. (2018). Mapping degradation signals and pathways in a eukaryotic N-terminome. Mol Cell *70*, 488-501.e5. 10.1016/j.molcel.2018.03.033.

7. Weill, U., Yofe, I., Sass, E., Stynen, B., Davidi, D., Natarajan, J., Ben-Menachem, R., Avihou, Z., Goldman, O., Harpaz, N., et al. (2018). Genome-wide SWAp-Tag yeast libraries for proteome exploration. Nat Methods *15*, 617–622. 10.1038/s41592-018-0044-9.

8. Kong, K.-Y.E., Fischer, B., Meurer, M., Kats, I., Li, Z., Rühle, F., Barry, J.D., Kirrmaier, D., Chevyreva, V., San Luis, B.-J., et al. (2021). Timer-based proteomic profiling of the ubiquitin-proteasome system reveals a substrate receptor of the GID ubiquitin ligase. Mol Cell *81*, 2460–2476. 10.1016/j.molcel.2021.04.018.

9. Kong, K.-Y.E., Reinbold, C., Knop, M., and Khmelinskii, A. (2023). Building yeast libraries to dissect terminal degrons with fluorescent timers. In Methods in Enzymology 10.1016/bs.mie.2023.02.012.

10. Yofe, I., Weill, U., Meurer, M., Chuartzman, S., Zalckvar, E., Goldman, O., Ben-Dor, S., Schütze, C., Wiedemann, N., Knop, M., et al. (2016). One library to make them all: Streamlining the creation of yeast libraries via a SWAp-Tag strategy. Nat Methods *13*, 371–378. 10.1038/nmeth.3795.
