# Supplementary material for: Orphan quality control by an SCF ubiquitin ligase directed to pervasive C-degrons
Source: Nat Commun. 2023 Dec 15;14:8363. doi: 10.1038/s41467-023-44096-z (PMC10724198; doi:10.1038/s41467-023-44096-z)
Supplement: Supplementary file 3 — Description of Additional Supplementary Files [file 41467_2023_44096_MOESM3_ESM.pdf]

<u>**Description of Additional Supplementary Files**</u>

File Name: **Supplementary Data 1**

Description: MPS profiling of the tFT-X_{12} library; peptide sequences and corresponding PSIs for two technical replicates.

File Name: **Supplementary Data 2**

Description: Targeted SGA screens for UPS factors involved in turnover of tFT-ΦN constructs**;** $\log_2$ fold changes in sfGFP intensity and mCherry/sfGFP ratios, and corresponding p-values, between each UPS mutant and a wild type control.

File Name: **Supplementary Data 3**

Description: MPS profiling of C-terminal capping libraries for tFT-tagged IN1, IN2, LN1, LN3, VN2, R1-R5, W1-W3, Atg1$^{-12–-1}$ and Rpa12$^{-12–-1}$ constructs in wild type and mutant (*das1*Δ or *doa10*Δ) backgrounds; peptide sequences and corresponding PSIs.

File Name: **Supplementary Data 4**

Description: MPS profiling of saturation mutagenesis libraries for tFT-tagged IN1, IN2, LN1, LN3, VN2, R1-R5, W1-W3, Atg1$^{-12–-1}$ and Rpa12$^{-12–-1}$ constructs in wild type and mutant (*das1*Δ or *doa10*Δ) backgrounds; peptide sequences and corresponding PSIs.

File Name: **Supplementary Data 5**

Description: MPS profiling of the tFT-X_{12} degron library; peptide sequences and corresponding PSIs across technical replicates and genetic backgrounds.

File Name: **Supplementary Data 6**

Description: MPS profiling of the yeast C-terminome library; peptide sequences and corresponding PSIs across technical replicates and genetic backgrounds.

File Name: **Supplementary Data 7**

Description: Primers, oligonucleotide pools and degenerate oligonucleotide sequences used in this study.
