# Supplementary material for: Orphan quality control by an SCF ubiquitin ligase directed to pervasive C-degrons
Source: Nat Commun. 2023 Dec 15;14:8363. doi: 10.1038/s41467-023-44096-z (PMC10724198; doi:10.1038/s41467-023-44096-z)
Supplement: Supplementary file 11 — Reporting Summary [file 41467_2023_44096_MOESM11_ESM.pdf]

Corresponding author(s): Khmelinskii, Anton

Last updated by author(s): Nov 23, 2023

Reporting Summary

Nature Portfolio wishes to improve the reproducibility of the work that we publish. This form provides structure for consistency and transparency in reporting. For further information on Nature Portfolio policies, see our Editorial Policies and the Editorial Policy Checklist.

Statistics

For all statistical analyses, confirm that the following items are present in the figure legend, table legend, main text, or Methods section.

n/a	Confirmed	
☐	☒	The exact sample size (*n*) for each experimental group/condition, given as a discrete number and unit of measurement
☐	☒	A statement on whether measurements were taken from distinct samples or whether the same sample was measured repeatedly
☐	☒	The statistical test(s) used AND whether they are one- or two-sided<br>*Only common tests should be described solely by name; describe more complex techniques in the Methods section.*
☒	☐	A description of all covariates tested
☐	☒	A description of any assumptions or corrections, such as tests of normality and adjustment for multiple comparisons
☐	☒	A full description of the statistical parameters including central tendency (e.g. means) or other basic estimates (e.g. regression coefficient) AND variation (e.g. standard deviation) or associated estimates of uncertainty (e.g. confidence intervals)
☐	☒	For null hypothesis testing, the test statistic (e.g. F, t, r) with confidence intervals, effect sizes, degrees of freedom and P value noted<br>*Give P values as exact values whenever suitable.*
☒	☐	For Bayesian analysis, information on the choice of priors and Markov chain Monte Carlo settings
☒	☐	For hierarchical and complex designs, identification of the appropriate level for tests and full reporting of outcomes
☐	☒	Estimates of effect sizes (e.g. Cohen's *d*, Pearson's *r*), indicating how they were calculated

Our web collection on statistics for biologists contains articles on many of the points above.

Software and code

Policy information about availability of computer code

Data collection	FACSDiva v9.0.1 on a FACSAria III SORP cell sorter (BD Biosciences)<br>FACSDiva v9.0.1 on a LSRFortessa SORP flow cytometer (BD Biosciences)<br>SparkControl v2.3 on a Spark plate reader (Tecan)<br>ImageLab Touch v3.0.1.14 on a ChemiDoc MP imaging system (Bio-Rad)<br>PhenoBooth v2.21.1117.1 on a PhenoBooth imaging system (Singer Instruments)<br>Leica Application Suite Advanced Fluorescence v2.7.3.9723 on a TCS SP5 microscope (Leica)
Data analysis	FACSDiva v9.0.1 (BD Biosciences)<br>bcl2fastq conversion software v2.20 (Illumina)<br>FastQC v0.11.9<br>FastQ Screen v0.13<br>Cutadapt v4.0<br>PEAR v0.9.11<br>UMI-Tools v1.1.2<br>Fiji/ImageJ v2.14.0/1.54f<br>Saccharomyces Genome Database (https://www.yeastgenome.org/)<br>Microsoft Excel (Microsoft)<br>R v4.0.3 with the following packages (tensorflow_2.9.0, keras_2.8.0, readxl_1.4.0, iml_0.11.1, ggrepel_0.9.1, protr_1.6-3, factoextra_1.0.7, venn_1.11, ggseqlogo_0.1, dummies_1.5.6, cowplot_1.1.1, ggpubr_0.4.0, Peptides_2.4.4, rstatix_0.7.0, stringr_1.4.1, stringi_1.7.6, dplyr_1.0.7, limma_3.46.0, ggplot2_3.3.6, reshape2_1.4.4, reshape_0.8.9, Rcpp_1.0.8.3, lattice_0.20-45, tidyr_1.1.4, listenv_0.8.0, png_0.1-7, assertthat_0.2.1, zeallot_0.1.0, digest_0.6.29, utf8_1.2.2, parallelly_1.32.1, R6_2.5.1, cellranger_1.1.0, plyr_1.8.7, backports_1.4.1,

pillar_1.8.0, tfruns_1.5.1, rlang_1.0.2, rstudioapi_0.14, data.table_1.14.2, whisker_0.4, car_3.1-1, Matrix_1.4-1, checkmate_2.0.0, reticulate_1.24, munsell_0.5.0, broom_1.0.1, compiler_4.0.3, base64enc_0.1-3, pkgconfig_2.0.3, globals_0.16.1, Metrics_0.1.4, tidyselect_1.1.2, tibble_3.1.6, codetools_0.2-18, fansi_0.5.0, future_1.28.0, withr_2.5.0, grid_4.0.3, jsonlite_1.8.0, gtable_0.3.1, lifecycle_1.0.1, DBI_1.1.3, magrittr_2.0.1, scales_1.2.1, cli_3.2.0, carData_3.0-5, ggsignif_0.6.4, ellipsis_0.3.2, admisc_0.26, generics_0.1.3, vctrs_0.3.8, prediction_0.3.14, tools_4.0.3, glue_1.6.0, purrr_0.3.4, abind_1.4-5, parallel_4.0.3, colorspace_2.0-2)

The pipeline for processing of MPS profiling data is available at https://github.com/Khmelinskii-Lab/Das1_C-degrons/tree/main/NGSpipe2go-MPSprofiling. The pipeline for downstream analysis and data visualization is available at https://github.com/Khmelinskii-Lab/Das1_C-degrons.

For manuscripts utilizing custom algorithms or software that are central to the research but not yet described in published literature, software must be made available to editors and reviewers. We strongly encourage code deposition in a community repository (e.g. GitHub). See the Nature Portfolio guidelines for submitting code & software for further information.

Data

Policy information about availability of data

All manuscripts must include a data availability statement. This statement should provide the following information, where applicable:

- Accession codes, unique identifiers, or web links for publicly available datasets
- A description of any restrictions on data availability
- For clinical datasets or third party data, please ensure that the statement adheres to our policy

The sequencing data generated in this study have been deposited in the Gene Expression Omnibus under accession code GSE246422. Processed MPS profiling data (peptide sequences and PSIs) are provided as Supplementary Data. The input data necessary for downstream analysis and data visualization are available at https://figshare.com/s/4c576dfd79e031878584. Source data are provided with this paper.

Research involving human participants, their data, or biological material

Policy information about studies with human participants or human data. See also policy information about sex, gender (identity/presentation), and sexual orientation and race, ethnicity and racism.

Reporting on sex and gender	Not applicable
Reporting on race, ethnicity, or other socially relevant groupings	Not applicable
Population characteristics	Not applicable
Recruitment	Not applicable
Ethics oversight	Not applicable

Note that full information on the approval of the study protocol must also be provided in the manuscript.

Field-specific reporting

Please select the one below that is the best fit for your research. If you are not sure, read the appropriate sections before making your selection.

☒ Life sciences ☐ Behavioural & social sciences ☐ Ecological, evolutionary & environmental sciences

For a reference copy of the document with all sections, see nature.com/documents/nr-reporting-summary-flat.pdf

Life sciences study design

All studies must disclose on these points even when the disclosure is negative.

Sample size	In MPS profiling experiments, library complexity was ensured by analyzing a number of cells at least 50-100 fold larger than the number of sequence variants in a library. Two to three technical replicates corresponding to independent sorting and sequencing of the same yeast library were analyzed. MPS profiling of C-terminal capping and saturation mutagenesis libraries was performed once, as all variants of each peptide could be directly compared. SGA screens were performed in four technical replicates (four colonies of the same strain) placed next to each other on the screen plates. All other experiments were performed with at least two biological replicates, i.e., independent clones isolated from a single transformation, as is standard in the field.
Data exclusions	No data were excluded.
Replication	Experiments were replicated the indicated number of times. None of the presented experimental results were not reproducible. The neural network was trained on replicate 1 of the tFT-X12 library. Its performance was evaluated on replicate 1, replicate 2 and on the average of replicates 1 and 2 with similar outcomes.
Randomization	No randomization was performed due to small sample size.

Blinding	No blinding was performed since the small sample groups have to be defined during data analysis. Determination was significance was based only on automated procedures. For flow cytometry, fluorescence microscopy and immunoblotting, blinding was not done as the samples were prepared and analyzed by the same researcher in small batches.

Reporting for specific materials, systems and methods

We require information from authors about some types of materials, experimental systems and methods used in many studies. Here, indicate whether each material, system or method listed is relevant to your study. If you are not sure if a list item applies to your research, read the appropriate section before selecting a response.

Materials & experimental systems

n/a	Involved in the study	
☐	☒	Antibodies
☐	☒	Eukaryotic cell lines
☒	☐	Palaeontology and archaeology
☒	☐	Animals and other organisms
☒	☐	Clinical data
☒	☐	Dual use research of concern
☒	☐	Plants

Methods

n/a	Involved in the study	
☒	☐	ChIP-seq
☐	☒	Flow cytometry
☒	☐	MRI-based neuroimaging

Antibodies

Antibodies used	Mouse anti-GFP (1:2000 dilution, 11814460001, Roche, clone number 7.1 and 13.1, RRID:AB_390913)<br>Mouse anti-Pgk1 (1:1000 dilution, 459250, Thermo Fisher Scientific, clone number 22C5D8, RRID:AB_2532235)<br>Goat HRP-conjugated anti-mouse (1:5000 dilution, G-21040, Thermo Fisher Scientific, RRID:AB_2536527)
Validation	All antibodies are commercially available and tested by the manufacturers.<br><br>Validation of the mouse anti-GFP (1:2000 dilution, 11814460001, Roche, clone number 7.1 and 13.1), information from the manufacturer:<br>Anti-GFP was obtained by immunizing mice with partially purified recombinant Aequorea victoria GFP as immunogen. Spleen cells were then fused with myeloma cells to create a variety of hybridoma clones. Hybridoma supernatants were screened for binding to the immunogen and specifically to highly purified recombinant GFP. Hybridomas secreting monoclonal antibodies specific for GFP were isolated and cloned by limiting dilution. Monoclonal antibodies were further screened for performance in western blot and immunoprecipitation applications using GFP fusion proteins. Anti-GFP antibody clones 7.1 and 13.1 were purified to >95% purity as determined by SDS-PAGE and HPLC analyses, then blended and lyophilized in phosphate-buffered saline in the presence of the protein stabilizer gelatin.<br><br>Validation of the mouse anti-Pgk1 (1:1000 dilution, 459250, Thermo Fisher Scientific, clone number 22C5D8):<br>Species Reactivity: Yeast. Published Species: Fungi, Yeast, Insect, Human, Mouse. Host/Isotype: Mouse / IgG1, kappa. Class: Monoclonal. Clone: 22C5D8. Immunogen: Full-length native S. cerevisiae pGK protein (purified). Purification: purified. RRID AB_2532235. This product reacts with Saccharomyces cerevisiae PGK1 - predicted molecular weight: 45 kDa. Purity >95% by SDS-PAGE.

Eukaryotic cell lines

Policy information about [cell lines and Sex and Gender in Research]

Cell line source(s)	All yeast strains used in this work are listed in Supplementary Table 7 and are derivatives of BY4741, Y8205, Y7092, ESM356-1, or PJ69-4A.
Authentication	Yeast genome manipulations (gene tagging and gene deletion) were verified by immunoblotting, diagnostic PCR or sequencing as appropriate.
Mycoplasma contamination	Not applicable
Commonly misidentified lines (See ICLAC register)	Not applicable

Flow Cytometry

Plots

Confirm that:

- [x] The axis labels state the marker and fluorochrome used (e.g. CD4-FITC).
- [x] The axis scales are clearly visible. Include numbers along axes only for bottom left plot of group (a 'group' is an analysis of identical markers).
- [ ] All plots are contour plots with outliers or pseudocolor plots.
- [x] A numerical value for number of cells or percentage (with statistics) is provided.

Methodology

Sample preparation	Yeast strains were grown in synthetic complete medium overnight to saturation, diluted into fresh medium to 0.2 OD600 and further grown for ~5 h to 0.8 OD600.
Instrument	Single cell fluorescence intensities were measured on a LSRFortessa SORP flow cytometer (BD Biosciences) using a 561 nm laser for mCherry excitation, a 600 nm long pass mirror and a 610/20 nm band pass filter for mCherry detection, a 488 nm laser for sfGFP excitation, a 505 nm long pass mirror and a 530/30nm band pass filter for sfGFP detection.<br>Fluorescence activated cell sorting was performed on a FACSAria III SORP cell sorter (BD Biosciences) with a 70 µm nozzle (70 psi) using a 561 nm laser for mCherry excitation, a 600 nm long pass mirror and a 610/20 nm band pass filter for mCherry detection, a 488 nm laser for sfGFP excitation, a 505 nm long pass mirror and a 530/30nm band pass filter for sfGFP detection.
Software	FACSDiva v9.0.1 (BD Biosciences) and Microsoft Excel (Microsoft)
Cell population abundance	Clonal yeast strains, not applicable.
Gating strategy	Measurements were gated for single cells with a sfGFP fluorescence intensity above the maximum intensity of a non-fluorescent control strain.

- [x] Tick this box to confirm that a figure exemplifying the gating strategy is provided in the Supplementary Information.
